# Cancer Burden on Piecemeal Endoscopic Resection of Early Adenocarcinoma in Barrett's Oesophagus Correlates With the Risk of Neoplastic Recurrence

**DOI:** 10.1002/ueg2.70140

**Published:** 2025-10-29

**Authors:** Grace J. Hattersley, Andreas V. Hadjinicolaou, Andrea Sorge, Daniel Conceicao, Sally Pan, Vijay Sujendran, Andrea Brown, Philip Kaye, Pradeep Mundre, Jacobo Ortiz‐Fernández‐Sordo, Massimiliano di Pietro

**Affiliations:** ^1^ School of Clinical Medicine University of Cambridge Cambridge UK; ^2^ Early Cancer Institute Department of Oncology University of Cambridge Cambridge UK; ^3^ Department of Gastroenterology Cambridge University Hospitals NHS Foundation Trust Cambridge UK; ^4^ Department of Pathophysiology and Transplantation University of Milan Milan Italy; ^5^ Department of Gastroenterology Instituto Português de Oncologia de Lisboa Francisco Gentil Lisbon Portugal; ^6^ Cambridge Oesophago‐Gastric Center Cambridge University Hospitals NHS Foundation Trust Cambridge UK; ^7^ Department of Gastroenterology Bradford Teaching Hospital Bradford UK; ^8^ Department of Histopathology Nottingham University Hospitals Nottingham UK; ^9^ NIHR Nottingham Digestive Diseases Biomedical Research Unit Nottingham University Hospitals Nottingham UK

**Keywords:** barretts oesophagus, EMR, endoscopic resection, ESD, oesophageal adenocarcinoma

## Abstract

**Background and Study Aims:**

Endoscopic resection (ER) is curative for early‐stage oesophageal adenocarcinoma (OAC) without high‐risk features. Piecemeal endoscopic mucosal resection (pEMR) prevents assessment of lateral margins, complicating risk estimation for neoplastic recurrence. We investigated the risk factors for residual and recurrent OAC post‐pEMR.

**Methods:**

We performed a longitudinal study of two independent patient cohorts: the test cohort who underwent piecemeal or en‐bloc ER (*n* = 140) and the validation cohort who underwent pEMR only (*n* = 89). Inclusion criteria were: OAC stage T1a or low‐risk T1b, no lympho‐vascular invasion, and R0 resection. The primary outcome was residual OAC at first post‐ER endoscopy, and secondary outcomes were residual neoplasia (high‐grade dysplasia and/or OAC), recurrence of neoplasia at any post‐ER endoscopy, and remission of neoplasia, dysplasia and metaplasia at most recent endoscopy.

**Results:**

In the test cohort, the incidence of neoplastic recurrence was higher in patients treated with pEMR (*n* = 54, 49%) versus en‐bloc ER (*n* = 7, 23%) (*p =* 0.021). The percentage of pEMR specimens with OAC was an independent risk factor for residual OAC at the first post‐pEMR endoscopy (OR for a 10% increase = 1.24, CI = 1.03–1.51, *p* = 0.025). A 50% cut‐off of pEMR specimens with OAC was optimal to predict residual OAC (specificity = 0.68, sensitivity = 0.63). Rates of residual (*p* = 0.039) and recurrent (*p* = 0.0052) OAC were higher when > 50% of pEMR specimens were involved by OAC. In the validation cohort, recurrent OAC was also more frequent when cancer burden was > 50% (*p* = 0.013).

**Conclusions:**

High cancer burden on pEMR specimens correlates with the risk of residual OAC. Post‐pEMR site check before endoscopic ablation is recommended if more than 50% of pEMR specimens show OAC.

## Key Summary

- Summarize the established knowledge on this subject.
  ◦ Endoscopic resection (ER) is the standard curative treatment for early‐stage oesophageal adenocarcinoma (OAC), with piecemeal resection often performed for lesions larger than 15 mm without suspicion of submucosal invasion
  ◦ Piecemeal mucosal resection (pEMR) prevents confident assessment of lateral margins, making estimation of the risk of residual disease difficult
  ◦ Use of a piecemeal resection technique has been shown to correlate with the risk of recurrence of OAC
  ◦ Current guidelines recommend ablation of remaining Barrett's oesophagus post‐ER; however, ablation of residual undetected flat cancer can reduce chances of cure
- What are the significant and/or new findings of this study?
  ◦ Patients treated with pEMR have increased rates of recurrent neoplasia compared with those treated with en‐bloc resection
  ◦ The percentage of specimens containing cancer is an independent risk factor for residual and recurrent OAC following pEMR
  ◦ In two independent cohorts, patients with OAC in more than 50% of pEMR specimens showed increased rates of residual and recurrent OAC over follow‐up
  ◦ A cut‐off of 50% of pEMR specimens with OAC can be used to risk stratify patients and inform which patients should receive more careful follow up prior to ablation of the remaining Barrett's epithelium

## 1 Introduction

There were an estimated 85,700 global cases of oesophageal adenocarcinoma (OAC) in 2020 [1]. This global burden has been increasing steadily since the 1970s, with a particular prevalence in male populations in the Western world, including Europe, North America, and Australia [2]. Indeed, in these areas, the incidence of OAC has surpassed that of oesophageal squamous cell carcinoma.

OAC typically develops on the background of Barrett's oesophagus (BO) [3]. While unselected screening is not typically indicated, in certain high‐risk groups such as patients with gastro‐oesophageal reflux disease (GORD) and additional risk factors including white race, male sex and obesity, targeted screening for BO may be considered [4, 5, 6]. For patients with diagnosed BO, endoscopic surveillance using high‐definition systems with mapping biopsies according to the Seattle protocol is currently recommended by many professional guidelines, with the frequency dependent on the length of the BO segment. If visible abnormalities are detected, targeted biopsies of the most suspicious part of the lesion should be taken. Overall, the goal of endoscopic surveillance is the detection of early‐stage low‐risk OAC, which is more amenable to endoscopic treatment.

T1a OAC with invasion confined to the muscularis mucosae is an absolute indication for endoscopic resection (ER). There is also a relative indication for ER in low‐risk T1b OAC, if invasion is limited to the superficial submucosa, differentiation is good or moderate and there is no evidence of lympho‐vascular invasion, because of the increasing evidence of low incidence of metastatic disease [5, 6]. At this early stage, ER is considered curative, provided the resection margins are histologically free of disease (R0), and is equally as effective as oesophagectomy while being associated with fewer adverse events [7]. Ablation therapy is subsequently performed post‐ER to eradicate the remaining BO epithelium and to reduce the risk of metachronous disease [8].

Endoscopic mucosal resection (EMR) is the recommended ER technique for early‐stage OAC of less than 20 mm in diameter and for larger BO‐associated flat dysplastic lesions [5]. Given the typical size of an EMR specimen is 15 mm, a piecemeal EMR (pEMR) is often required to ensure complete lesion resection. For early‐stage OAC that is larger than 20 mm in diameter or lesions with suspected superficial submucosal invasion, endoscopic submucosal dissection (ESD) is recommended. However, previous studies have shown that expert endoscopists poorly predict submucosal invasion based on endoscopic features and suboptimally agree on the Paris classification [9, 10]. Limited studies have been performed comparing the efficacy of these two techniques for OAC [11]; however, it is of note that ESD is associated with longer procedural time and the absolute requirement of general anaesthetic [12].

The goal of an ER is to remove all cancerous tissue and to clear the field of visible surrounding dysplasia. In BO, this can be difficult to fully recognise as the epithelium can bear foci of flat intramucosal cancer. Therefore, ER poses the risk of residual or recurrent disease due to dysplastic margins being overlooked, leading to inadequate resection [7]. pEMR may have a higher risk of such recurrence as it is not possible to assess lateral margins histologically post‐resection, and stratification for the risk of residual and recurrent adenocarcinoma is more challenging compared to en‐bloc ER [13, 14]. Importantly, performing ablation therapy in the presence of residual OAC can jeopardise outcomes by burying disease foci and enabling deeper proliferation of dysplastic lesions [15, 16]. However, no studies to date have developed a method to inform the risk of residual OAC post‐pEMR.

The primary aim of this study was to identify risk factors for residual OAC at the first post‐pEMR endoscopy in our patient cohort. The secondary aims were to generate a clinical template that could be used to risk stratify patients treated with pEMR for OAC.

## 2 Materials and Methods

### 2.1 Study Design and Participants

We conducted a longitudinal cohort study of patients with early‐stage oesophageal adenocarcinoma (OAC) who were treated with endoscopic resection (ER). The test cohort comprised of patients treated at Cambridge University Hospitals (CUH) between 29/03/2006 and 02/11/2023, recruited through the ethically approved Cambridge registry study (LREC01/149). We included 140 patients, of which 110 patients were treated with piecemeal endoscopic mucosal resection (pEMR), 22 with en‐bloc endoscopic mucosal resection (eEMR) and 8 with en‐bloc endoscopic submucosal dissection (ESD). Fewer patients were included in the en‐bloc (eEMR or ESD) group compared to the piecemeal group due to the clinical practice pattern during the study period, where pEMR was the predominant technique for early OAC. The validation independent cohort included 89 patients treated with pEMR at Nottingham University Hospitals (NUH) between 02/01/2012 and 26/07/2024 (*n* = 57) and at Bradford Teaching Hospitals (BTH) between 01/05/2014 and 13/11/2015 (*n* = 32). All patients in the test and validation cohorts provided informed consent to participate in the research.

### 2.2 Inclusion and Exclusion Criteria

Inclusion criteria were: intramucosal (T1a) OAC or low‐risk submucosal (T1bsm1) OAC (invasion into the submucosal layer limited to 500 microns, good to moderate differentiation); no evidence of lympho‐vascular invasion (LVI‐); clear resection margins on histological evaluation (R0 resection), defined as free vertical margins for pEMR and free vertical and radial margins for en‐bloc ER; adequate post‐ER follow‐up. In the test cohort, adequate post‐ER follow‐up was defined as a diagnostic endoscopy with biopsies, or repeat ER in case of visible lesions, prior to ablation therapy. This inclusion criterion was restricted to patients in the test cohort because performing a site‐check prior to radiofrequency ablation (RFA) is routine practice at CUH. Conversely, ablation was typically performed at the immediate post‐pEMR endoscopy in the validation cohort, with biopsies only taken in cases of suspicious lesions or, at BTH, if the lateral pEMR margin was positive for invasive cancer. Patients in the validation cohort who did not have biopsies from the immediate post‐pEMR endoscopy were excluded from relevant analysis if they went on to have recurrence of OAC or HGD as it would not be possible to distinguish between residual and recurrent OAC.

Exclusion criteria were: high‐grade dysplasia (HGD) only; high‐risk T1b OAC (poor differentiation/grade 3 or T1bsm2–3); R1 resection; and inadequate information reported post‐ER. Overall, 8 patients were excluded from the piecemeal cohort due to an R1 resection, with deep margins involved. 10 patients treated with en‐bloc resection were excluded due to R1 resection, caused by lateral margin involvement with deep margins clear in 8 cases, and deep margin involvement with lateral margins clear in 2 cases.

### 2.3 Endoscopic Procedures

Endoscopic mucosal resection (EMR) was typically performed under conscious sedation, while ESD was always performed under general anaesthesia. From 2020 at NUH, procedures were performed under deep propofol sedation or general anaesthetic at the discretion of the anaesthetist.

pEMR was completed using a Duette device (Cook medical) with prior marking by a snare tip. The decision to perform submucosal injection and endoscopic mucosal resection (EMR) margin coagulation was at the discretion of the treating physician. As part of this study, we were unable to ascertain completion of the marked area resection. Following resection, pEMR specimens were individually pinned on cardboard.

En‐bloc resection was performed either with the EMR Duette kit, or with ESD, which was introduced at CUH in 2017. Decision for EMR versus ESD was taken based on early cancer lesion factors, with ESD preferred for sessile lesions (Paris 0‐Is), lesions with depression (Paris 0‐IIc), or lesions that could not be sucked in the Duette cap. At NUH, ESD was also preferred in patients with a history of prior endotherapy. Given that the cohort partly preceded the most recent European Society of Gastrointestinal Endoscopy (ESGE) guidelines, the size of the lesion was typically not a determinant for treatment decision.

Reporting of the ER specimen was performed according to the Vienna classification. The pathologist reported the number of specimens involved by OAC in the case of pEMR. We calculated cancer burden, the percentage of pEMR specimens involved by OAC, as the number of piecemeal resections with OAC on histology divided by the total number of piecemeal resections received.

Following a potentially curative ER at CUH, patients received a site check at 2–3 months prior to ablation therapy such as RFA. During this procedure, biopsies would be taken on the EMR scar, or a subsequent ER would be performed in case of clear evidence of residual neoplasia. Indication for RFA was flat Barrett's with no evidence of visible neoplastic lesion at the post‐ER endoscopic follow‐up. In the validation cohort, a pre‐ablation site check was not typically performed, and RFA would be completed immediately post‐pEMR endoscopy.

### 2.4 Outcomes

The primary outcome was histological diagnosis of residual OAC in biopsies taken from the first post‐ER endoscopy. Secondary outcomes were: any residual neoplasia (OAC and/or HGD) in biopsies taken from the first post‐ER endoscopy; histological evidence of recurrence of OAC identified in biopsies taken from any post‐ER endoscopy (including residual disease identified at the first post‐ER endoscopy); histological evidence of recurrence of neoplasia; and complete remission of OAC, dysplasia (OAC and/or HGD and/or LGD) and metaplasia (OAC and/or HGD and/or LGD and/or intestinal metaplasia (IM)) in biopsies taken from the most recent endoscopy.

### 2.5 Analysis

Descriptive statistics are expressed as medians (interquartile range) or frequency of observations with percentages. Patients in the CUH cohort who did not have alcohol consumption, ever smoker status, body mass index (BMI), or endoscopic lesion size reported, and patients in the validation cohort who either had no biopsies taken at the most recent endoscopy or where pathology at the most recent endoscopy was not available, were excluded from the relevant analyses. For discrete variables, chi‐squared tests were applied to contingency tables where all observations were greater than 5, otherwise Fisher's exact test was applied. For continuous variables, the Shapiro‐Wilk test was applied to assess for normality, followed by the Mann‐Whitney test for non‐parametric distributions or the Student's T‐test for parametric distributions. Kaplan‐Meier analysis with a log‐rank test and a univariable Cox hazard model was performed to compare recurrence over time. The percentage of pEMR specimens with OAC was calculated as the number of pEMR specimens with histological evidence of OAC divided by the number of pEMR specimens assessed histologically. Firth's penalised logistic regression models were generated to evaluate possible risk factors for primary and secondary outcomes. Variables included in the model were selected based on previous reports in the literature, and all variables were included in the multivariable model, except where they were based on a different cohort size (BMI and lesion size) or accounted for by an alternative variable (number of pEMR specimens with OAC and total number of pEMR specimens). Youden's index was applied to receiver operating characteristics (ROC) curves to identify the optimal cut‐off of pEMR specimens with OAC for residual and recurrent OAC detection. *p* values less than 0.05 were considered statistically significant. Power analysis showed that the validation cohort would require a total sample size of 79 to detect the same difference in recurrence of OAC by pEMR cut‐off that was observed in the test cohort at *α* of 0.05 and power of 0.8 (1‐β).

All statistical analysis was performed using R for Statistical Computing v4.4.1 [R core team]. Kaplan Meier analysis was completed using the survival package and ROC analysis was completed using the pROC package. Figures were plotted using the ggplot2 and survminer packages.

## 3 Results

### 3.1 The Incidence of Neoplastic Recurrence is Higher in Patients Treated With Piecemeal Compared to En‐Bloc Resection

**FIGURE 1:**
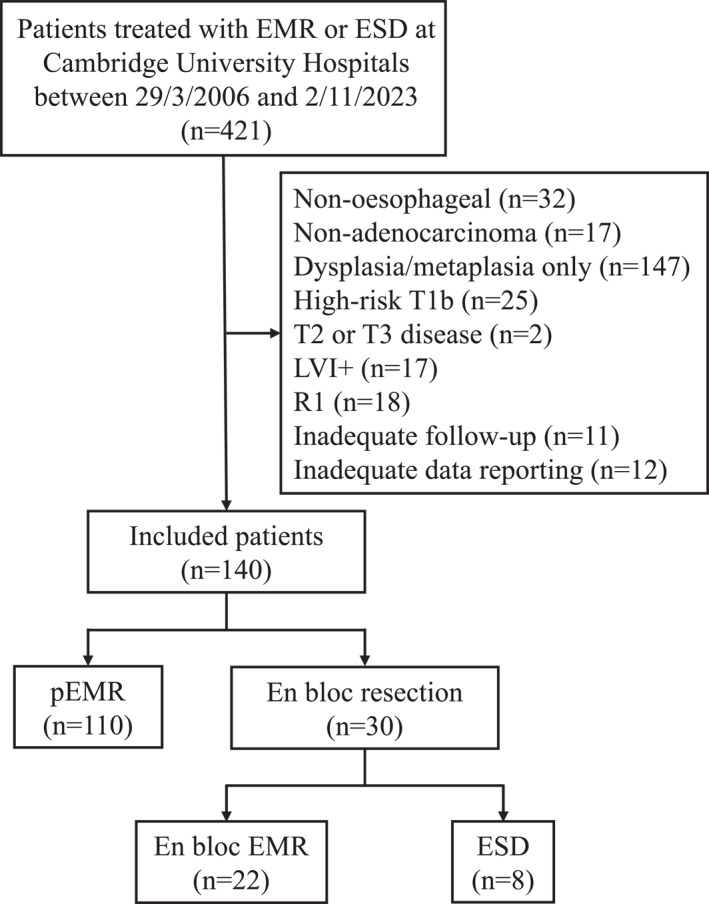
Flow chart demonstrating included and excluded patients.

421 patients with records of an oesophageal ER performed at Cambridge University Hospitals were assessed for inclusion in the study (Figure 1). After exclusion criteria were applied, we included 140 individuals who received ER for early‐stage oesophageal adenocarcinoma (OAC) (Table 1). 110 individuals were treated with piecemeal endoscopic mucosal resection (pEMR), and 30 individuals were treated with en‐bloc ER (EMR *n* = 22; ESD *n* = 8) (Supporting Information S1: Table 1). Baseline cohort characteristics did not differ between piecemeal and en‐bloc groups, except for body mass index (BMI), which was higher in individuals treated with en‐bloc ER (*p* = 0.019). However, the percentage of overweight patients did not differ between ER types (*p* = 1). Lesions resected by pEMR were on average larger (*p* = 0.042) and the underlying OAC was more commonly of poor differentiation (*p* = 0.0058), while lesions resected en‐bloc were more often sessile, as characterised by the Paris Classification (*p =* 0.002).

**TABLE 1:** Cohort characteristics by the type of resection, pEMR or en‐bloc ER (en‐bloc EMR and ESD).

	Total cohort (*n* = 140)	pEMR (*n* = 110)	En‐bloc ER (*n* = 30)	*p*‐value
Age	68.5 (64–76)	69 (64.25–77)	68 (64–73)	0.26
Sex (male)	112 (80%)	89 (81%)	23 (77%)	0.8
BMI^a^	28.9 (26–31.25)	28.6 (25.85–30.65)	30.9 (27.5–35.9)	**0.019**
Overweight (BMI > 25)^a^	110 (84%)	86 (83%)	24 (86%)	1
Time to first follow‐up (months)	2 (1–2)	2 (1–2)	2 (2–3.75)	0.083
Duration of follow‐up (months)	43.5 (20–70.25)	42.5 (20.5–69.25)	49.5 (20.25–73.25)	0.77
Ever smoker^b^	78 (70%)	58 (68%)	20 (74%)	0.74
Alcohol excess (> 14 units per week)^c^	15 (14%)	13 (16%)	2 (8%)	0.51
Lesion size (mm)^d^	15 (10–20)	20 (10–20)	10 (8–20)	**0.042**
Paris classification^e^				
0‐IIa	63 (45%)	51 (46%)	12 (40%)	0.68
0‐IIb	48 (34%)	40 (36%)	8 (27%)	0.44
0‐IIc	3 (2%)	3 (3%)	0 (0%)	1
0‐IIa‐c	5 (4%)	5 (5%)	0 (0%)	0.58
0‐Is	14 (10%)	6 (5%)	8 (27%)	**0.002**
0‐Isp	1 (1%)	1 (1%)	0 (0%)	1
Stage (T1bsm1)	7 (5%)	5 (5%)	2 (7%)	0.64
Differentiation (poor)	7 (5%)	6 (5%)	1 (3%)	**0.0058**
Worst histology at first follow‐up				
OAC	26 (19%)	20 (18%)	6 (20%)	1
HGD	20 (14%)	20 (18%)	0 (0%)	**0.0074**
LGD	14 (10%)	10 (9%)	4 (13%)	0.5
IM	60 (43%)	47 (43%)	13 (43%)	1
Residual disease				
OAC	26 (19%)	20 (18%)	6 (20%)	1
Neoplasia	46 (33%)	40 (36%)	6 (20%)	0.14
Recurrence within 1 year				
OAC	36 (26%)	29 (26%)	7 (23%)	0.92
Neoplasia	56 (40%)	49 (45%)	7 (23%)	0.058
Recurrence anytime				
OAC	37 (26%)	30 (27%)	7 (23%)	0.84
Neoplasia	61 (44%)	54 (49%)	7 (23%)	**0.021**
Worst histology at last follow‐up				
OAC	11 (8%)	10 (9%)	1 (3%)	0.46
HGD	3 (2%)	3 (3%)	0 (0%)	1
LGD	10 (7%)	8 (7%)	2 (7%)	1
IM	38 (27%)	29 (26%)	9 (30%)	0.87

*Note:* Data are *n* (%) or median (IQR). Bold indicates *p* < 0.05. Residual disease refers to the presence of the defined pathology at the first post‐ER follow‐up. Recurrent disease refers to the presence of the defined pathology at any, including the first, post‐ER follow‐up. Neoplasia is defined as HGD and/or OAC.

^a^ Based on a total cohort size of 131.

^b^ Based on a total cohort size of 112.

^c^ Based on a total cohort size of 105.

^d^ Based on a total cohort size of 107.

^e^ Based on a total cohort size of 134.

Altogether, 37 patients exhibited OAC on biopsies taken at any post‐ER, of which 26 (70%) occurred at the immediate post‐ER endoscopy (Table 1). Moreover, neoplasia (HGD and/or OAC) was identified in biopsies taken at any post‐ER endoscopy in 61 individuals, of which 46 (75%) were diagnosed at the first post‐ER endoscopy. Correspondingly, most of these cases (84%) of recurrence of neoplasia occurred within the first 3 months following initial endoscopic resection. Of note, 11/46 cases of residual neoplasia were detected at first follow‐up through non‐targeted biopsies performed in the absence of a visible lesion on endoscopy. Such cases would not have been detectable in the validation cohort because it is routine practice at these hospitals to perform ablation therapy at the immediate post‐ER endoscopy in the absence of a visible lesion.

**FIGURE 2:**
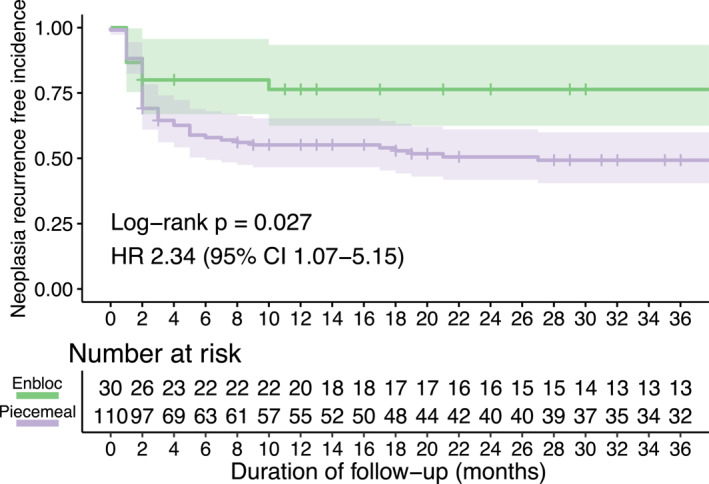
Neoplasia recurrence‐free survival of patients post‐ER by resection type over a 3‐year period. Time measured from the date of first endoscopic resection. Recurrence of neoplasia refers to histological evidence of OAC and/or HGD at any time, including the first post‐ER endoscopy. Shaded regions represent 95% confidence intervals; crosses denote censored patients. The hazard ratio (HR) and *p* value calculated using a log rank test represent the comparison between individuals treated with pEMR and en‐bloc ER.

Patients treated with pEMR demonstrated a higher rate of recurrence of neoplasia over the total follow‐up period compared with individuals treated with en‐bloc ER (*p* = 0.021, Table 1). This difference was confirmed by Kaplan Meier analysis, which showed that the cumulative incidence of recurrence of neoplasia was higher in patients treated with pEMR compared with en‐bloc ER over a 3‐year period (*p* = 0.027, HR 2.34, 95% CI 1.07–5.15; Figure 2). We hypothesised that this could be due to dysplastic margins being overlooked and thus left in situ because of the lack of circumferential incision in pEMR. Since pEMR prevents assessment of lateral resection margins, we investigated alternative factors that could be used to identify patients at risk of residual OAC post‐pEMR.

### 3.2 The Percentage of pEMR Specimens With OAC is an Independent Risk Factor for Residual OAC at the First Post‐pEMR Endoscopy

We generated a multivariable logistic regression model to identify clinical factors that influenced the risk of residual OAC post‐pEMR (Table 2). This revealed that the percentage of pEMR specimens with OAC at the initial resection (cancer burden) independently predicted the risk of residual OAC at the first post‐pEMR endoscopy (*p* = 0.025). On univariable analysis, we identified that this was likely due to an increase in the absolute number of pEMR specimens with OAC (*p* = 0.042) rather than due to a reduction in the number of pEMR specimens taken (*p* = 0.32). Similarly, the percentage of pEMR specimens with OAC at the initial resection was an independent risk factor for recurrence of OAC identified at any post‐pEMR endoscopy (*p* = 0.011; Supporting Information S1: Table 2). However, the percentage of pEMR specimens with OAC was not a risk factor for residual neoplasia at the first post‐pEMR endoscopy (OAC and/or HGD: *p* = 0.36), or recurrence of neoplasia identified at any (including the first) post‐pEMR endoscopic follow‐up (*p* = 0.17). Moreover, remission of OAC (*p* = 0.38), neoplasia (*p* = 0.87) and dysplasia (OAC, HGD and LGD; *p* = 0.96) at the most recent endoscopy was not associated with the percentage of pEMR specimens with OAC.

**TABLE 2:** Univariable and multivariable logistic regression models for residual OAC at the first post‐pEMR endoscopy.

	Univariable		Multivariable	
	OR (95% CI)	*p*‐value	OR (95% CI)	*p*‐value
Age at first resection^a^	0.97 (0.92–1.03)	0.32	0.96 (0.9–1.01)	0.13
Alcohol abuse (> 14 units/per week)^a^	1.65 (0.38–5.77)	0.47	2.19 (0.46–8.85)	0.3
BMI	0.95 (0.87–1.04)	0.29		
Differentiation^a^	1.27 (0.53–3.09)	0.6	1.19 (0.48–3.05)	0.71
Lesion size	1.03 (0.97–1.09)	0.37		
Number of piecemeal resections	1.09 (0.91–1.28)	0.32		
Number of resections with OAC	1.26 (1.01–1.59)	**0.042**		
Percentage of resections with OAC^a^, ^b^	1.23 (1.02–1.48)	**0.028**	1.24 (1.03–1.51)	**0.025**
Sex^a^	0.58 (0.19–1.89)	0.35	0.77 (0.24–2.85)	0.68
Ever smoker^a^	0.78 (0.29–2.06)	0.61	0.89 (0.3–2.61)	0.82
Stage^a^	3.61 (0.57–20.04)	0.16	4.83 (0.61–34.17)	0.13

*Note:* Bold values indicate *p* < 0.05.

^a^ Inclusion in the multivariable model. BMI and lesion size could not be included in the multivariable model due to incomplete information for all patients. Number of piecemeal resections and number of resections with OAC are not included in the multivariable model as they are accounted for by the percentage of resections with OAC.

^b^ Odds ratio for a 10% increase in the number of pEMR specimens with cancer on histological investigation.

Given the association between the percentage of samples with OAC and residual and recurrent cancer risk, we generated receiver operating characteristics (ROC) curves to identify a threshold of pEMR specimens with OAC above which patients were more likely to experience residual and recurrent cancer. This revealed that a 53.5% cut‐off of pEMR specimens with OAC at the initial resection maximised sensitivity and specificity for residual (Supporting Information S1: Figure 1A) and recurrent (Supporting Information S1: Figure 1B) OAC detection. Clinically, we determined that this would correlate to a > 50% cut‐off, given that a minimum of 23 specimens would be necessary to distinguish a 50% cut‐off from a 53.5% cut‐off.

### 3.3 Patients With an OAC Burden of More Than 50% Show Increased Rates of Residual and Recurrent OAC Post‐pEMR

**FIGURE 3:**
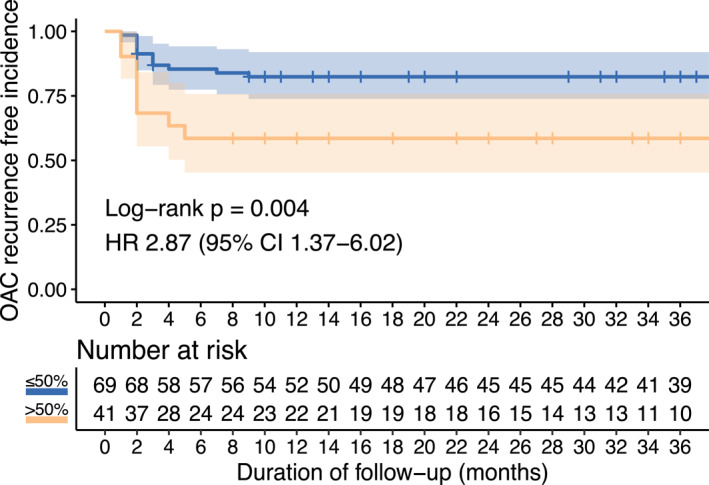
OAC recurrence‐free survival of patients post‐pEMR by percentage of pEMR specimens affected by OAC. Time measured from the date of first endoscopic resection. Recurrence of OAC is defined as detection of OAC on histology at any time, including the first post‐ER endoscopy. Shaded regions represent 95% confidence intervals; crosses denote censored patients. The hazard ratio (HR) and *p* value calculated using a log rank test represents the comparison between individuals with > 50% and ≤ 50% of pEMR specimens with OAC.

To investigate whether this cut‐off of pEMR specimens with OAC could be used to stratify patients, we subdivided the pEMR cohort (*n* = 110) into patients with more than 50% cancer burden (*n* = 41) and those with less than 50% cancer burden (*n* = 69) (Table 3). Baseline characteristics did not differ between these two groups. We found that patients with more than 50% specimens involved by OAC at the initial pEMR showed an increased rate of residual OAC at first post‐pEMR endoscopy (*p* = 0.039), recurrence of OAC (*p* = 0.011) and neoplasia (OAC and/or HGD, *p* = 0.038) at 1 year, and recurrence of OAC (*p* = 0.0052) and neoplasia (*p* = 0.012) at any post‐pEMR endoscopy. This was corroborated by Kaplan Meier analysis, which identified an increased incidence of OAC recurrence in patients with OAC burden > 50% over a 3‐year period (Figure 3, *p* = 0.004). The rate of remission at most recent follow up of OAC (*p* = 0.17), neoplasia (*p* = 0.31), dysplasia (OAC and/or HGD and/or LGD: *p* = 0.4) and metaplasia (OAC and/or HGD and/or LGD and/or IM: *p* = 1) were not affected by this cut‐off.

**TABLE 3:** Cohort characteristics by percentage cut‐off.

Test cohort (*n* = 110)		≤ 50% OAC burden (*n* = 69)	> 50% OAC burden (*n* = 41)	*p*‐value
Age	69 (64.25–77)	68 (64–75)	72 (67–77)	0.14
Sex (male)	89 (81%)	57 (83%)	32 (78%)	0.74
Duration of follow‐up (months)	42.5 (20.5–69.25)	48 (24–73)	36 (17–57)	0.086
Stage (T1bsm1)	5 (5%)	2 (3%)	3 (7%)	0.36
Differentiation (poor)	6 (5%)	2 (3%)	4 (10%)	0.19
Worst histology at first follow‐up				
OAC	20 (18%)	8 (12%)	12 (29%)	**0.039**
HGD	20 (18%)	13 (19%)	7 (17%)	1
LGD	10 (9%)	10 (14%)	0 (0%)	**0.013**
IM	47 (43%)	31 (45%)	16 (39%)	0.68
Residual disease				
OAC	20 (18%)	8 (12%)	12 (29%)	**0.039**
Neoplasia	40 (36%)	21 (30%)	19 (46%)	0.14
Recurrence within 1 year				
OAC	29 (26%)	12 (17%)	17 (41%)	**0.011**
Neoplasia	49 (45%)	25 (36%)	24 (59%)	**0.038**
Recurrence anytime				
OAC	30 (27%)	12 (17%)	18 (44%)	**0.0052**
Neoplasia	54 (49%)	27 (39%)	27 (66%)	**0.012**
Worst histology at last follow‐up				
OAC	10 (9%)	4 (6%)	6 (15%)	0.17
HGD	3 (3%)	2 (3%)	1 (2%)	1
LGD	8 (7%)	5 (7%)	3 (7%)	1
IM	29 (26%)	20 (29%)	9 (22%)	0.56
Remission				
OAC	100 (91%)	65 (94%)	35 (85%)	0.17
Neoplasia	97 (88%)	63 (91%)	34 (83%)	0.31
Dysplasia	89 (81%)	58 (84%)	31 (76%)	0.4
Metaplasia	60 (55%)	38 (55%)	22 (54%)	1

**Validation cohort (*n* = 89)**		**≤** **50% OAC burden (*n* = 60)**	**>** **50% OAC burden (*n* = 29)**	*p* **‐value**
Age	70 (62–76)	70 (62–76.25)	69 (63–76)	0.87
Sex (male)	72 (81%)	48 (80%)	24 (83%)	0.98
Duration of follow‐up (months)	43.5 (12.75–70)	45 (13–69)	32 (9–69)	0.61
Stage (T1bsm1)	7 (8%)	2 (3%)	5 (17%)	**0.035**
Differentiation (poor)	9 (10%)	8 (13%)	1 (3%)	0.15
Worst histology at first FUP^a^				
OAC	11 (13%)	6 (11%)	5 (17%)	0.59
HGD	23 (27%)	14 (25%)	9 (31%)	0.7
LGD	6 (7%)	3 (5%)	3 (10%)	0.4
IM	28 (33%)	20 (35%)	8 (28%)	0.65
Residual disease^a^				
OAC	11 (14%)	6 (12%)	5 (18%)	0.73
Neoplasia	34 (44%)	20 (41%)	14 (50%)	0.59
Recurrence within 1 year				
OAC	14 (16%)	7 (12%)	7 (24%)	0.23
HGD	36 (40%)	21 (35%)	15 (52%)	0.2
Recurrence anytime				
OAC	21 (24%)	9 (15%)	12 (41%)	**0.013**
HGD	44 (49%)	26 (43%)	18 (62%)	0.15
Histology at last FUP^b^				
OAC	7 (9%)	3 (6%)	4 (15%)	0.21
HGD	6 (8%)	5 (9%)	1 (4%)	0.66
LGD	4 (5%)	1 (2%)	3 (12%)	0.1
IM	17 (22%)	12 (23%)	5 (19%)	0.96
Remission^b^				
OAC	72 (91%)	50 (94%)	22 (85%)	0.21
Neoplasia	66 (84%)	45 (85%)	21 (81%)	0.89
Dysplasia	62 (78%)	44 (83%)	18 (69%)	0.27
Metaplasia	45 (57%)	32 (60%)	13 (50%)	0.53

*Note:* Top panel, test cohort, derived from patients treated at a single centre; bottom panel, validation cohort, derived from patients treated at two independent centres. Data are *n* (%) or median (IQR). Bold indicates *p* < 0.05. Residual disease refers to the presence of the defined pathology at the first post‐ER follow‐up. Recurrent disease refers to the presence of the defined pathology at any, including the first, post‐ER follow‐up. Remission refers to the absence of the defined pathology at final follow‐up. Neoplasia is defined as HGD and/or OAC. Dysplasia is defined as LGD and/or HGD and/or OAC. Metaplasia is defined as IM and/or LGD and/or HGD and/or OAC.

^a^ Based on a total cohort size of 86.

^b^ Based on a total cohort size of 79.

We further confirmed that patients who show OAC in more than 50% of pEMR specimens have a higher risk of disease recurrence in an independent patient cohort. This validation cohort comprised of 89 patients treated at two independent institutions (Table 3). 29/89 patients had pEMR OAC burden > 50%, and 60/89 patients had pEMR OAC burden ≤ 50%. Baseline cohort characteristics were similar, except patients with a higher OAC burden were more commonly of the T1bsm1 stage (*p* = 0.035). Importantly, we identified that there was an increased frequency of OAC recurrence in patients where more than 50% of pEMR specimens had OAC (*p* = 0.013). This consistency of our cut‐off across multiple independent centres supports its use to stratify patients as high‐risk for disease recurrence.

## 4 Discussion

To the best of our knowledge, this study is the first to investigate risk factors for residual OAC after pEMR. We showed that there was an increased rate of HGD recurrence post‐pEMR compared with en‐bloc resection modalities, including en‐bloc EMR and ESD. The percentage of cancerous samples was an independent risk factor for residual‐OAC post‐pEMR, with a 50% cut‐off of pEMR specimens with OAC representing the best threshold to identify patients at the highest risk of residual and recurrent cancer.

Percentage cancer burden on pEMR represents a novel approach to risk stratifying patients for residual OAC and can be used to complement other models and techniques that inform risk after successful endoscopic treatment. For instance, a model to predict visible dysplastic recurrence following complete eradication of Barrett's oesophagus with OAC or dysplasia has previously been developed [17]. The risk factors included in the model are new visible lesions during treatment, higher number of ER treatments, male sex, increasing Barrett's oesophagus length, HGD or cancer at baseline, and younger patient age. While this model enables a long‐term follow‐up plan to be tailored to an individual patient's risk factors, the use of percentage cancer burden may be more easily implemented. This is because it is based on a single intuitive variable rather than multiple patient and treatment related factors. Hence, endoscopists can quickly evaluate the percentage of pEMR specimens with OAC prior to the immediate post‐ER endoscopy to allow rapid decision making on further therapy and follow‐up.

Evaluation of lesion size represents a further technique by which endoscopists risk stratify patients post‐ER, given that it is known to be associated with increased risk of recurrence [18]. While increasing the pEMR cancer burden could be considered a surrogate of this, using the percentage of pEMR specimens with OAC as a risk stratification tool has several advantages. First, sizing lesions in BO can be challenging due to the field of flat dysplasia. Second, the percentage cancer burden can also reflect the endoscopist radicality in the resection. A high percentage of pEMR specimens with OAC could either reflect a limited endoscopic resection of a smaller lesion or a large neoplastic lesion. In both scenarios, the theoretical risk of residual neoplasia is higher, and thus using percentage cancer burden as a risk stratification tool encompasses additional risk factors not accounted for by lesion size. Of note, we did not identify any correlation between the percentage of pEMR specimens with OAC and the absolute number of piecemeal resections (*R* ^2^ = 0.016, *p* = 0.092), further supporting this variable as an independent risk factor for residual and recurrent OAC.

The results of our study also have important implications in determining the optimal timing of ablation therapy following ER. Current clinical guidelines recommend ablation of the remaining Barrett's epithelium after treatment of dysplasia or OAC with endoscopic resection. The most common ablation modality is radiofrequency ablation (RFA), which uses high temperature to a depth of 500 microns to destroy the oesophageal epithelium. This is an approved treatment modality for dysplastic BO, whereas RFA is contraindicated in the presence of intramucosal adenocarcinoma due to the risk of invasion beyond 500 microns. In this setting, RFA would cause burial of the neoplastic columnar epithelium and would enable deep proliferation that evades surveillance [15, 16, 19]. However, limited research has been performed to date to identify the optimal timing of RFA after endoscopic resection, or to assess if there is a benefit in delaying RFA to prevent such subsquamous metaplasia. The EUROII study, which investigated the efficacy of RFA after EMR for T1a OAC, delayed ablation therapy until at least two post‐EMR site checks with mapping biopsies which showed no evidence of cancer were performed. This study showed a very high rate of resolution of neoplasia and BO of 92% and 87%, respectively [8]. Our findings suggest that patients treated with pEMR, and in particular those who have a greater than 50% cancer burden, are at higher risk for inadequate resection and recurrent dysplasia and OAC. Therefore, we recommend that such high‐risk patients should not receive RFA at the immediate post‐pEMR endoscopy to reduce the risk of burying residual OAC. Caution should generally be applied to patients treated for OAC with pEMR before starting RFA, and any visible flat lesions should be biopsied or resected.

A higher rate of dysplastic recurrence has previously been reported in patients treated for OAC with EMR compared to ESD, in which the en‐bloc rate was lower in patients treated with EMR, and in patients treated with a piecemeal ER compared to en‐bloc ER [18, 20, 21]. We argue that this higher rate of recurrence could be attributed to the difficulty in ensuring adequate resection of the lesion margin. Other studies comparing ESD with EMR have found no difference in the recurrence rate [11, 22, 23]. Our results do not advocate against pEMR as, in our cohort, careful site check and repeat EMR if required achieved remission of OAC in more than 90% of cases. There is an ongoing randomised clinical trial comparing the efficacy of ESD and EMR for the treatment of BO‐related neoplasia with a primary outcome related to recurrent or residual neoplasia in the two groups at 12 months (NCT05276791). The results of this trial will be useful to validate our findings. However, this RCT will recruit patients with non‐invasive neoplastic lesions, which was outside the remit of our study.

Our study has several limitations. While patients in the Cambridge cohort were recruited prospectively, retrieval of lesion size information and Paris classification required retrospective review of electronic healthcare records. As such, lesion size information and Paris classification are missing for some patients, and we could not retrieve sufficient data to include these variables in the logistic regression model. The clinical risk predictor was developed based on data from a single centre, and from a patient cohort treated over a 17‐year period; hence, clinical guidelines and treatment recommendations are likely to have changed throughout the study. Our centre performed fewer en‐bloc resections compared with pEMR, and a limited number of ESD procedures could be included. This limits the generalizability of findings comparing en‐bloc to piecemeal resection and prevents robust subgroup analysis between en‐bloc EMR and ESD. Despite this, we were able to detect the previously established pattern of increased rates of recurrence of OAC post‐piecemeal compared with en‐bloc resection, aligning our study with those generated from larger patient cohorts [18, 20, 21]. Additionally, while considerably larger than our en‐bloc group, our piecemeal cohort is still limited in size, and we therefore cannot exclude the risk that the logistic regression model developed in this cohort is overfitted. However, the impact of this was mitigated through validation of the strongest predictor in a multicentre cohort. Finally, power calculation to determine the minimum validation cohort size to detect the same difference in recurrence of OAC assumed that the ratio of patients with more than 50% of pEMR specimens affected by OAC was the same as in the test cohort. However, data analysis revealed that the validation cohort had a greater proportion of patients with less than 50% cancer burden. Hence, post hoc power calculations revealed a power of 0.76, despite the minimum sample size of 79 patients being reached.

In summary, our study provides a novel, simple and clinically applicable parameter for risk stratification of patients with low‐risk early OAC treated with pEMR and advocates careful assessment of these patients post‐ER prior to starting ablation treatment.

## Funding

M.d.P. is funded by the Medical Research Council and received additional funds by the Cancer Research UK Cambridge Centre [CTRQQR‐2021\100,012]. This research received infrastructure support from the Experimental Cancer Medicine Centre and the NIHR Cambridge Biomedical Research Centre [BRC‐1215‐20,014]. A.S. and D.C. were funded by the United European Gastroenterology visiting fellowship program. The views expressed are those of the authors and not necessarily those of the NIHR or the Department of Health and Social Care.

## Ethics Statement

The main cohort was recruited at Cambridge University Hospitals as part of the ethically approved registry study (LREC01/149). For the validation cohort, the study was assessed by local institutional review boards at Bradford Teaching Hospitals and Nottingham University Hospitals and was deemed not to require ethics approval.

## Consent

Patients at Cambridge University Hospital signed informed consent forms to participate in the research. Patients at Bradford Teaching hospitals and Nottingham University Hospitals consented to the use of data as part of ethically approved studies on the standard endoscopy consent form.

## Conflicts of Interest

The authors declare no conflicts of interest.

## Permission to Reproduce Material From Other Sources

The authors have nothing to report.

## Supporting information


Supporting Information S1

## Data Availability

Anonymised clinical data will be made available upon reasonable request.
