# Supplementary material for: Cancer Burden on Piecemeal Endoscopic Resection of Early Adenocarcinoma in Barrett's Oesophagus Correlates With the Risk of Neoplastic Recurrence
Source: United European Gastroenterol J. 2025 Oct 29;13(9):1703–14. doi: 10.1002/ueg2.70140 (PMC12605956; doi:10.1002/ueg2.70140)
Supplement: Supplementary file 1 — Supporting Information S1 [file UEG2-13-1703-s001.docx]

**Supplementals**

Supplementary Table 1

|  | En-bloc cohort (n=30) | eEMR (n=22) | ESD (n=8) | P-value | |
| --- | --- | --- | --- | --- | --- |
| Age | 68 (64-73) | 68 (64-72.75) | 67.5 (63-75) | 0.87 | |
| Sex (Male) | 23 (77%) | 16 (73%) | 7 (88%) | 0.64 | |
| BMI^*^ | 30.9 (27.5-35.9) | 29.75 (26.15-32.65) | 35.225 (30.55-39) | **0.021** | |
| Overweight (BMI > 25)^*^ | 24 (86%) | 16 (80%) | 8 (100%) | 0.29 | |
| Time to first follow-up (months) | 2 (2-3.75) | 2 (1.25-2.75) | 4 (2-6) | 0.079 | |
| Duration of follow-up (months) | 49.5 (20.25-73.25) | 55.5 (34-96.5) | 19 (12.5-29.25) | **0.012** | |
| Ever smoker^**^ | 20 (74%) | 16 (80%) | 4 (57%) | 0.33 | |
| Alcohol excess (>14 units per week)^***^ | 2 (8%) | 2 (11%) | 0 (0%) | 1 | |
| Lesion size (mm) ^†^ | 10 (8-20) | 9 (6-10) | 30 (15-41.25) | **0.00081** | |
| Paris Classification ^† †^ | | | | | |
| 0-IIa | 12 (40%) | 11 (50%) | 1 (12%) | 0.099 | |
| 0-IIb | 8 (27%) | 6 (27%) | 2 (25%) | 1 | |
| 0-Is | 8 (27%) | 3 (14%) | 5 (62%) | **0.016** | |
| Stage (T1bsm1) | 2 (7%) | 1 (5%) | 1 (12%) | 0.47 | |
| Differentiation (Poor) | 1 (3%) | 1 (5%) | 0 (0%) | 1 | |
| Worst histology at first follow-up | | | | |  |
| OAC | 6 (20%) | 6 (27%) | 0 (0%) | 0.16 | |
| LGD | 4 (13%) | 4 (18%) | 0 (0%) | 0.55 | |
| IM | 13 (43%) | 10 (45%) | 3 (38%) | 1 | |
| Residual disease | | | | | |
| OAC | 6 (20%) | 6 (27%) | 0 (0%) | 0.16 | |
| Neoplasia | 6 (20%) | 6 (27%) | 0 (0%) | 0.16 | |
| Recurrence within one year | | | | |  |
| OAC | 7 (23%) | 7 (32%) | 0 (0%) | 0.14 | |
| Neoplasia | 7 (23%) | 7 (32%) | 0 (0%) | 0.14 | |
| Recurrence anytime | | | | |  |
| OAC | 7 (23%) | 7 (32%) | 0 (0%) | 0.14 | |
| Neoplasia | 7 (23%) | 7 (32%) | 0 (0%) | 0.14 | |
| Worst histology at last follow-up | | | | |  |
| OAC | 1 (3%) | 1 (5%) | 0 (0%) | 1 | |
| LGD | 2 (7%) | 2 (9%) | 0 (0%) | 1 | |
| IM | 9 (30%) | 6 (27%) | 3 (38%) | 0.67 | |

**Supplementary Table 1: Cohort characteristics by type of en-bloc resection, en-bloc EMR or ESD.** Data are n (%) or median (IQR). Bold indicates *p*<0.05. Residual disease refers to the presence of the defined pathology at the first post-ER follow-up. Recurrent disease refers to the presence of the defined pathology at any, including the first, post-ER follow-up. Neoplasia is defined as HGD and/or OAC. No lesions in the en-bloc cohort were of Paris Classification 0-IIc, 0-IIa-c or 0-Isp. There were no instances where the worst histology at either first or last follow-up was HGD. *Based on a total cohort size of 28. **Based on a total cohort size of 27. ***Based on a total cohort size of 24. ^†^Based on a total cohort size of 22. ^††^Based on a total cohort size of 28.

Supplementary Table 2

|  | **Univariable** | | **Multivariable** | |
| --- | --- | --- | --- | --- |
|  | **OR (95% CI)** | **P** | **OR (95% CI)** | **P** |
| Residual neoplasia (OAC and/or HGD) at the first post-pEMR follow-up | | | | |
| Age at first resection^*^ | 0.97 (0.92-1.01) | 0.099 | 0.96 (0.92-1) | 0.082 |
| Alcohol abuse (>14 units/per week) ^*^ | 0.54 (0.13-1.78) | 0.32 | 0.58 (0.13-2.01) | 0.4 |
| BMI | 0.99 (0.92-1.06) | 0.74 |  |  |
| Differentiation^*^ | 1.12 (0.56-2.25) | 0.75 | 1 (0.49-2.05) | 0.99 |
| Lesion size (mm) | 1.04 (0.99-1.1) | 0.11 |  |  |
| Number of piecemeal resections | 1.06 (0.92-1.24) | 0.39 |  |  |
| Number of resections with OAC | 1.11 (0.91-1.37) | 0.3 |  |  |
| Percentage of resections with OAC^*†^ | 1.06 (0.91-1.23) | 0.45 | 1.07 (0.92-1.25) | 0.36 |
| Sex^*^ | 0.71 (0.27-1.87) | 0.48 | 0.83 (0.32-2.27) | 0.71 |
| Ever smoker^*^ | 0.72 (0.33-1.56) | 0.41 | 0.86 (0.39-1.91) | 0.72 |
| Stage^*^ | 1.25 (0.2-6.73) | 0.79 | 1.49 (0.22-8.73) | 0.66 |
| Residual dysplasia (OAC and/or HGD and/or LGD) at the first post-pEMR follow-up | | | | |
| Age at first resection^*^ | 0.98 (0.94-1.02) | 0.3 | 0.98 (0.93-1.02) | 0.27 |
| Alcohol abuse (>14 units/per week) ^*^ | 0.52 (0.14-1.65) | 0.28 | 0.61 (0.16-1.99) | 0.41 |
| BMI | 0.95 (0.87-1.02) | 0.14 |  |  |
| Differentiation^*^ | 1.36 (0.7-2.7) | 0.37 | 1.29 (0.65-2.59) | 0.47 |
| Lesion size (mm) | 1.04 (0.99-1.09) | 0.15 |  |  |
| Number of piecemeal resections | 0.99 (0.85-1.14) | 0.88 |  |  |
| Number of resections with OAC | 0.98 (0.8-1.2) | 0.87 |  |  |
| Percentage of resections with OAC^*†^ | 0.98 (0.85-1.13) | 0.76 | 0.98 (0.84-1.13) | 0.77 |
| Sex^*^ | 0.9 (0.35-2.31) | 0.82 | 0.99 (0.39-2.6) | 0.98 |
| Ever smoker^*^ | 0.82 (0.39-1.73) | 0.6 | 0.92 (0.43-1.97) | 0.83 |
| Stage^*^ | 1.72 (0.32-10.72) | 0.52 | 1.95 (0.35-12.53) | 0.44 |
| Residual metaplasia (OAC and/or HGD and/or LGD and/or IM) at the first post-pEMR follow-up | | | | |
| Age at first resection^*^ | 1.06 (1-1.12) | 0.071 | 1.06 (0.99-1.13) | 0.076 |
| Alcohol abuse (>14 units/per week) ^*^ | 1.22 (0.26-11.86) | 0.83 | 1.39 (0.27-14.17) | 0.72 |
| BMI | 0.94 (0.85-1.05) | 0.23 |  |  |
| Differentiation^*^ | 1.13 (0.41-3.2) | 0.81 | 1.22 (0.44-3.48) | 0.7 |
| Lesion size (mm) | 1.03 (0.95-1.14) | 0.5 |  |  |
| Number of piecemeal resections | 0.93 (0.78-1.15) | 0.43 |  |  |
| Number of resections with OAC | 0.88 (0.69-1.18) | 0.35 |  |  |
| Percentage of resections with OAC^*†^ | 0.92 (0.75-1.15) | 0.46 | 0.89 (0.72-1.11) | 0.3 |
| Sex^*^ | 2.18 (0.58-7.24) | 0.23 | 2.08 (0.54-7.1) | 0.27 |
| Ever smoker^*^ | 0.96 (0.3-2.97) | 0.94 | 0.71 (0.2-2.3) | 0.57 |
| Stage^*^ | 1.61 (0.17-215.18) | 0.74 | 1.45 (0.12-207.14) | 0.8 |
| Recurrence of OAC at any (including the first) post-pEMR follow-up | | | | |
| Age at first resection^*^ | 1 (0.95-1.05) | 0.94 | 0.99 (0.94-1.04) | 0.65 |
| Alcohol abuse (>14 units/per week) ^*^ | 0.85 (0.2-2.87) | 0.81 | 1.29 (0.28-4.91) | 0.73 |
| BMI | 0.99 (0.91-1.06) | 0.72 |  |  |
| Differentiation^*^ | 1.37 (0.65-2.95) | 0.41 | 1.16 (0.53-2.61) | 0.71 |
| Lesion size (mm) | 1.06 (1-1.12) | **0.032** |  |  |
| Number of piecemeal resections | 1.13 (0.97-1.34) | 0.12 |  |  |
| Number of resections with OAC | 1.37 (1.1-1.77) | **0.0039** |  |  |
| Percentage of resections with OAC^*†^ | 1.24 (1.06-1.46) | **0.0074** | 1.23 (1.05-1.46) | **0.011** |
| Sex^*^ | 0.89 (0.33-2.64) | 0.83 | 1.27 (0.43-4.29) | 0.68 |
| Ever smoker^*^ | 0.42 (0.17-0.96) | **0.04** | 0.42 (0.17-1.01) | 0.054 |
| Stage^*^ | 1.94 (0.31-10.52) | 0.45 | 2.2 (0.28-14.72) | 0.42 |
| Recurrence of neoplasia (OAC and/or HGD) at any (including the first) post-pEMR follow-up | | | | |
| Age at first resection^*^ | 1 (0.96-1.04) | 0.9 | 0.99 (0.94-1.03) | 0.57 |
| Alcohol abuse (>14 units/per week) ^*^ | 0.45 (0.12-1.4) | 0.17 | 0.54 (0.14-1.79) | 0.32 |
| BMI | 0.96 (0.89-1.03) | 0.3 |  |  |
| Differentiation^*^ | 1.97 (1-4.04) | 0.051 | 1.71 (0.86-3.54) | 0.13 |
| Lesion size (mm) | 1.09 (1.03-1.16) | **0.002** |  |  |
| Number of piecemeal resections | 1.44 (1.15-1.88) | **0.00045** |  |  |
| Number of resections with OAC | 1.54 (1.18-2.17) | **0.0007** |  |  |
| Percentage of resections with OAC^*†^ | 1.11 (0.96-1.28) | 0.17 | 1.09 (0.94-1.27) | 0.24 |
| Sex^*^ | 1.07 (0.42-2.76) | 0.89 | 1.18 (0.46-3.14) | 0.73 |
| Ever smoker^*^ | 1.08 (0.51-2.27) | 0.84 | 1.24 (0.57-2.69) | 0.59 |
| Stage^*^ | 1.48 (0.28-9.21) | 0.64 | 1.42 (0.25-9.18) | 0.69 |
| Remission of OAC at the last post-pEMR follow-up | | | | |
| Age at first resection^*^ | 1.01 (0.94-1.08) | 0.68 | 1.02 (0.95-1.09) | 0.57 |
| Alcohol abuse (>14 units/per week) ^*^ | 3.24 (0.38-425.52) | 0.35 | 1.84 (0.18-249.16) | 0.66 |
| BMI | 1 (0.88-1.12) | 0.95 |  |  |
| Differentiation^*^ | 0.55 (0.16-1.74) | 0.31 | 0.73 (0.22-2.28) | 0.59 |
| Lesion size (mm) | 0.97 (0.91-1.04) | 0.35 |  |  |
| Number of piecemeal resections | 0.92 (0.77-1.18) | 0.43 |  |  |
| Number of resections with OAC | 0.83 (0.65-1.12) | 0.2 |  |  |
| Percentage of resections with OAC^*†^ | 0.87 (0.69-1.11) | 0.25 | 0.9 (0.7-1.15) | 0.38 |
| Sex^*^ | 1.23 (0.22-4.92) | 0.79 | 0.63 (0.08-3.01) | 0.59 |
| Ever smoker^*^ | 4.32 (1.12-23.7) | **0.033** | 3.61 (0.92-19.57) | 0.066 |
| Stage^*^ | 0.3 (0.05-3.17) | 0.27 | 0.26 (0.03-3.65) | 0.28 |
| Remission of neoplasia (OAC and/or HGD) at the last post-pEMR follow-up | | | | |
| Age at first resection^*^ | 1.01 (0.95-1.07) | 0.74 | 1.02 (0.96-1.09) | 0.45 |
| Alcohol abuse (>14 units/per week) ^*^ | 4.31 (0.51-563.69) | 0.22 | 2.91 (0.29-400.26) | 0.43 |
| BMI | 1.02 (0.92-1.13) | 0.7 |  |  |
| Differentiation^*^ | 0.66 (0.23-1.83) | 0.42 | 0.81 (0.27-2.32) | 0.7 |
| Lesion size (mm) | 0.95 (0.89-1.01) | 0.11 |  |  |
| Number of piecemeal resections | 0.81 (0.66-0.96) | **0.017** |  |  |
| Number of resections with OAC | 0.77 (0.6-0.98) | **0.036** |  |  |
| Percentage of resections with OAC^*†^ | 0.96 (0.78-1.2) | 0.72 | 0.98 (0.78-1.25) | 0.87 |
| Sex^*^ | 0.88 (0.16-3.31) | 0.86 | 0.38 (0.04-1.84) | 0.25 |
| Ever smoker^*^ | 3.92 (1.19-16.31) | **0.024** | 4.25 (1.19-20.23) | **0.025** |
| Stage^*^ | 0.17 (0.03-1.11) | 0.063 | 0.1 (0.01-0.83) | **0.034** |
| Remission of dysplasia (OAC and/or HGD and/or LGD) at the last post-pEMR follow-up | | | | |
| Age at first resection^*^ | 0.96 (0.91-1.02) | 0.18 | 0.97 (0.92-1.02) | 0.27 |
| Alcohol abuse (>14 units/per week) ^*^ | 2.2 (0.49-21.06) | 0.34 | 1.3 (0.26-12.9) | 0.77 |
| BMI | 1.06 (0.97-1.16) | 0.21 |  |  |
| Differentiation^*^ | 0.6 (0.25-1.4) | 0.24 | 0.66 (0.27-1.61) | 0.37 |
| Lesion size (mm) | 0.97 (0.92-1.03) | 0.28 |  |  |
| Number of piecemeal resections | 0.84 (0.7-0.99) | **0.038** |  |  |
| Number of resections with OAC | 0.85 (0.68-1.06) | 0.14 |  |  |
| Percentage of resections with OAC^*†^ | 0.97 (0.81-1.16) | 0.71 | 1 (0.84-1.22) | 0.96 |
| Sex^*^ | 0.73 (0.18-2.34) | 0.62 | 0.57 (0.12-1.98) | 0.4 |
| Ever smoker^*^ | 2.59 (1-7.19) | 0.05 | 2.71 (1.02-7.78) | **0.045** |
| Stage^*^ | 0.32 (0.06-2) | 0.2 | 0.34 (0.06-2.38) | 0.26 |
| Remission of metaplasia (OAC and/or HGD and/or LGD and/or IM) at the last post-pEMR follow-up | | | | |
| Age at first resection^*^ | 0.97 (0.93-1.01) | 0.13 | 0.97 (0.93-1.02) | 0.21 |
| Alcohol abuse (>14 units/per week) ^*^ | 1.34 (0.43-4.46) | 0.61 | 0.97 (0.3-3.34) | 0.96 |
| BMI | 1.03 (0.96-1.11) | 0.38 |  |  |
| Differentiation^*^ | 0.65 (0.33-1.28) | 0.22 | 0.68 (0.33-1.36) | 0.28 |
| Lesion size (mm) | 0.99 (0.94-1.03) | 0.55 |  |  |
| Number of piecemeal resections | 0.97 (0.83-1.12) | 0.67 |  |  |
| Number of resections with OAC | 0.98 (0.8-1.2) | 0.81 |  |  |
| Percentage of resections with OAC^*†^ | 0.98 (0.85-1.14) | 0.83 | 1.02 (0.88-1.18) | 0.81 |
| Sex^*^ | 0.89 (0.34-2.27) | 0.8 | 0.8 (0.3-2.09) | 0.66 |
| Ever smoker^*^ | 1.41 (0.67-2.99) | 0.37 | 1.48 (0.69-3.23) | 0.31 |
| Stage^*^ | 0.26 (0.03-1.47) | 0.13 | 0.31 (0.03-1.86) | 0.21 |

**Supplementary Table 2: Logistic regression model for residual neoplasia at the first post-pEMR endoscopy, recurrence of OAC or neoplasia identified at any future endoscopy and remission of OAC, neoplasia, dysplasia and metaplasia identified at most recent endoscopy.** *Inclusion in the multivariable model. BMI and lesion size could not be included in the multivariable model due to incomplete information for all patients. Number of piecemeal resections and number of resections with OAC are not included in the multivariable model as they are accounted for by the percentage of resections with OAC. ^†^Odds ratio for a 10% increase in the number of pEMR specimens with cancer on histological investigation.

Supplementary Figure 1


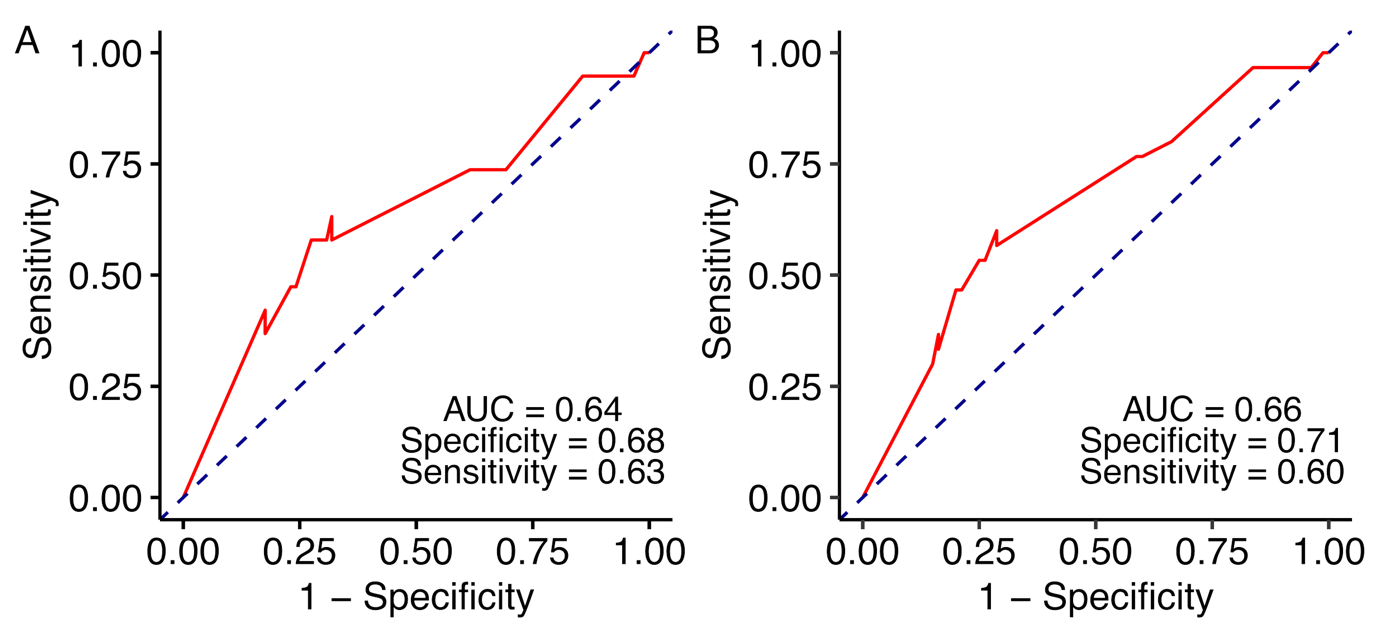


**Supplementary Figure 1: Receiver operating characteristics (ROC) curves.** (A) ROC curve for prediction of risk of residual OAC based on the percentage of pEMR specimens with OAC at initial resection. Sensitivity and specificity are indicated for a 53.5% cut-off. (B) ROC curve for prediction of risk of recurrent OAC based on the percentage of pEMR specimens with OAC at initial resection. Sensitivity and specificity are indicated for a 53.5% cut-off.
